# Modeling of the Gelation Process in Cellulose Aerogels

**DOI:** 10.1021/acs.biomac.4c01474

**Published:** 2025-03-03

**Authors:** Jannik Jarms, Nina H. Borzęcka, Bruno Serrador Goncalves, Kathirvel Ganesan, Barbara Milow, Ameya Rege

**Affiliations:** † Institute of Materials Research, German Aerospace Center (DLR), Linder Höhe, 51147 Cologne, Germany; ‡ Institute of Mechanics and Computational Mechanics, Gottfried Wilhelm Leibniz University Hannover, Appelstraße 9a, 30167 Hannover, Germany; ¶ Department of Inorganic and Materials Chemistry, University of Cologne, Greinstr. 6, 50939 Cologne, Germany; § Department of Mechanics of Solids, Surfaces & Systems, University of Twente, P.O. Box 217, 7500 AE Enschede, The Netherlands

## Abstract

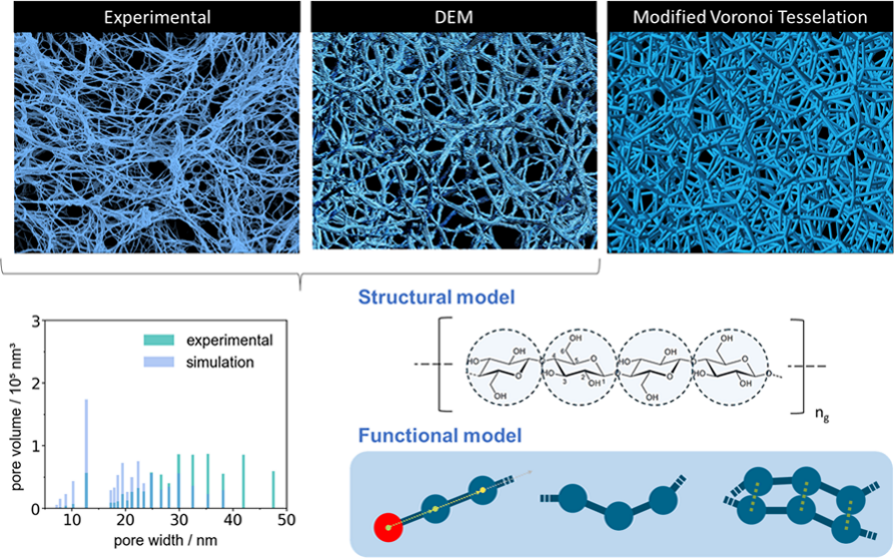

Cellulose aerogels are the most well-studied biopolymer-based systems in the literature, yet we lack a complete understanding of the underlying gelation mechanism, as well as that of the effect of solvent exchange on the topology of their network. This work presents a coarse-grained model describing the gelation kinetics in cellulose aerogel systems. A discrete element model is employed to generate the cellulose structure, and the solvents are modeled implicitly. Langevin dynamics is applied to solve the system of Newtonian equations. The model successfully generates the structure of the cellulose gel, hydrogel, alcogel, as well as aerogel. A model parameter sensitivity analysis is presented, and the results of the model are validated against the experimental data. The model provides insights into the mechanism of gelation while also shedding light on the morphological alterations resulting from the washing, solvent exchange, and drying steps.

## Introduction

Aerogels are nanostructured open-porous materials that are synthesized from various inorganic and organic sources. IUPAC recently listed aerogels among the top 10 emerging technologies in chemistry in 2022.^1^ This class of materials has attracted significant attention owing to their exceptionally low densities and thermal conductivity. A special class of these materials arises from biobased sources, predominantly from polysaccharides and proteins, and is gaining prominence owing to their sustainable, biocompatible, and recyclable characteristics.^2^ Among these, cellulose-based systems are the most well-studied ones.^3^

While several reports exist on cellulose aerogels, the mechanism of their gelation remains to be fully understood. For targeted reverse engineering of these materials, a well-informed correlation between the synthesis and process parameters and the structural and morphological features needs to be established. Here, theoretical and computational methods can prove to be useful. Rege et al.^4^ first proposed a constitutive model for describing cellulose aerogels. The model was based on the mechanics of the pore walls and was shown to be useful in predicting the mechanical structure–property relations. An alternative approach was proposed by Chandrasekaran et al.^5^ for modeling biopolymer aerogels and was based on the radical Voronoi method. In this model, a random closed pack of polydisperse spheres was generated, one that represented the pore volume distribution in the aerogels. Laguerre–Voronoi tessellations were generated on these spheres, and after eliminating these spheres, an open-porous cellular solid was presented and subsequently subjected to mechanical deformation. This model could also accurately predict the mechanical structure–property relations in aerogels and was more concrete in terms of prediction than the previously proposed constitutive model because there were no fitting parameters involved. However, as one can observe, the models proposed to date have dealt with generating or using the final morphology of the aerogels to study their mechanical behavior. These models do not account for mapping the network formation of the material and thus cannot be used for better understanding the gelation in such aerogel systems.

Biopolymer aerogels are prepared by using a synthesis route different from the standard sol–gel process. In more classical aerogels, such as silica-based ones, the starting blocks of the gel network are simple molecular units, namely, monomers, produced from the chosen precursor. The formation of the gel network from these simple monomers is known to be typically modeled using, e.g., aggregation algorithms.^6−8^ On the other hand, in the case of biopolymer systems, the starting blocks are macromolecular structures of the chosen material. The underlying process of network formation is different from that of silica aerogels. It becomes imperative to understand the gelation mechanism in such polymer-based systems, which begins with aggregation of the polymer chains, resulting in fibrillization and the resulting fibrils forming a 3D interconnected porous network, the gel.

To this end, we propose a new model for modeling gelation, solvent exchange, and drying in such cellulose aerogel systems. Our approach is modified from the previously proposed model for alginate gels by Depta et al.^9^ The model is based on the discrete element method and applies a coarse-grained molecular dynamics approach to solve the system of Newtonian equations.

## Materials and Experimental Methods

### Materials

For the purpose of this work, a commercially available cellulose powder was used. The cellulose was purchased from Sigma-Aldrich and extracted from cotton linters. It is described as a medium chain length cellulose with product number C6288. An average molecular weight distribution (*M*_n_) of 61,760 g mol^–1^ (DP = 180) reported in the literature was taken for analysis.^10^ Sodium hydroxide was obtained from J.T. Baker, urea from Sigma-Aldrich, and acetic acid (glacial) from VWR.

### Production of Cellulose Aerogel

Cellulose aerogel beads were produced by the method reported in the literature.^11^

**Figure 1:**
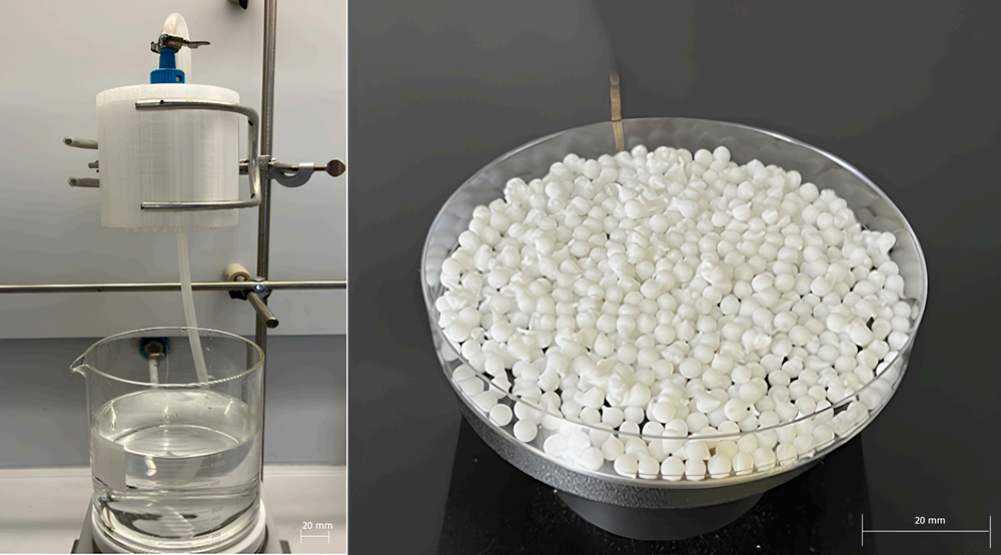
Illustrating the conventional multinozzle dropping setup (left) and aerogel beads (right).

The wet-gel beads were produced by a conventional dropping technique (see Figure 1). It is a multinozzle dropping setup having a nozzle diameter of 3 mm. The cellulose (7 g) in 100 g of NaOH-urea-water solution was dropped into a gelation bath containing 2 M of aqueous acetic acid. The wet-gel beads were formed after the complete diffusion of acid through the cellulose droplet. After a 30 min gelation period, the wet-gel beads were subsequently washed several times with water in order to neutralize the beads. Afterward, a stepwise solvent exchange with ethanol was performed. After the solvent exchange, the beads were dried under supercritical CO_2_ conditions using a HTPE-150p extractor. The drying process was conducted at 115 bar and 60 °C with an average CO_2_ mass flow of 22.5 kg h^–1^.

### Infrared Spectroscopy

The infrared (IR) analysis of the aerogel beads was done with the Bruker Tensor 27 using an attenuated total reflectance-Fourier transform infrared (ATR-FTIR) module. The FTIR measurement was done with a resolution of 4 cm^–1^ and 40 scans. The aim was to confirm that the prepared cellulose aerogels have no impurities.

### X-ray Diffraction

The X-ray diffraction (XRD) measurements of the aerogel beads were carried out on a Bruker D8 ADVANCE A25 diffractometer using Cu Kα radiation with a wavelength of λ = 1.54 Å. The spectra were recorded in a range between 5 and 80° (2θ) at a scan rate of 1° min^–1^. The standard parameters for the reflection mode were 35 kV and 30 mA. The aerogel beads were compressed and ground to a fine powder form using a mortar and pestle in order to employ them in the XRD measurements.

### Volume Shrinkage

The volume shrinkage (denoted as *V*_s_) of the cellulose aerogel beads was calculated that the beads had spherical geometries.
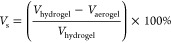
1

The volume of aerogel (*V*_aerogel_) and the volume of hydrogel (*V*_hydrogel_) were used in eq 1 to analyze the volume shrinkage.

### Densities and Porosity

The skeletal density was measured with helium gas, and the analysis was carried out in the AccuPyc II 1340, Micromeritics. For the measurement, cellulose aerogel beads were finely ground in a mortar, filled in the sample container, and subsequently compressed. The sample mass was measured beforehand. The volume of the sample was about 70% of that of the sample container. For each measurement, the sample in the chamber was purged 10 cycles with helium gas in order to remove the adsorbed atmospheric gas molecules. Average skeletal density was reported after 10 cycles of analysis during each measurement.

Envelope density analysis was performed with Geopyc 1360, Micromeritics, working with DryFlow as the enclosing medium around the sample. The precision cylinder used as a sample cell was filled with DryFlow up to 2.5 cm. The volume of a sample was around 25% compared to the DryFlow amount. 51 N of force was applied during the measurement. The envelope density was measured for 10 cycles during each measurement. This procedure was repeated twice for the cellulose aerogel beads.

The bulk density was determined to correspond to DIN EN ISO 60. Three measurements were performed for each sample.

The porosity (in %) was calculated from the skeletal density (ρ_s_) and envelope density (ρ_e_), using the following equation:
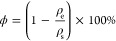
2

### Nitrogen Adsorption–Desorption Isotherm

The nitrogen adsorption–desorption technique was applied to determine the BET-specific surface area, pore volume, and pore-size distribution of the cellulose aerogel beads by TriStar II 3020 device, Micromeritics. Prior to the physisorption analysis, the beads were vacuum-dried using the VacPrep 061, Micrometrics instrument at 110 °C overnight. This ensures that the samples do not contain any water.

### Scanning Electron Microscopy

The morphology of the aerogel beads was determined via scanning electron microscopy (SEM) using an Ultra 55 microscope by Zeiss. For this procedure, a representative fraction of the sample was placed on a sample holder that was equipped with a carbon adhesive pad. The samples were sputtered with platinum for 100 s with a current of 21 mA. This resulted in a sputter coating thickness of ca. 10 nm. For the SEM analyses, a voltage of 3 kV was applied with a working distance of 8.7–9.1 mm.

## Computational Model

The gelation process in the studied cellulose gels is computationally followed by the coarse-grained molecular dynamics method (CGMD). The motion of cellulose chains, consisting of a given number of d-glucose molecules as a repeating unit, is described with the discrete element method (DEM), an approach for computing the motion of particles by solving Newton’s equation of motion.

The investigated system is implemented in the open-source DEM simulation framework MUSEN.^12^ The simulated cellulose system considers a cubic representative volume element (RVE) with a length of *l*_RVE_ = 100 nm for which cubic periodic boundary conditions are defined. With the simulation progress, one can follow the gelation process of cellulose chains within the RVE, which represents a given volume of an aqueous NaOH-urea solution. The developed gelation model is modified from the model reported by Depta et al.^9^ The computational approach consists of an ensemble of a structural model and a functional model, where the latter is subdivided into a diffusion model, a polymer bond model, and an interaction model. The gelation simulation of the cellulose chains is followed by washing and solvent exchange simulations of the obtained gel.

Within this section, the description of the computational approach is presented, including an overview of the CGMD and the DEM approach implementation for the cellulose system, the description of the functional model subcomponents (structural, functional, and gelation model), the choice of model parameters, the simulation procedure, as well as details concerning their implementation.

### Structural Model

**Figure 2:**
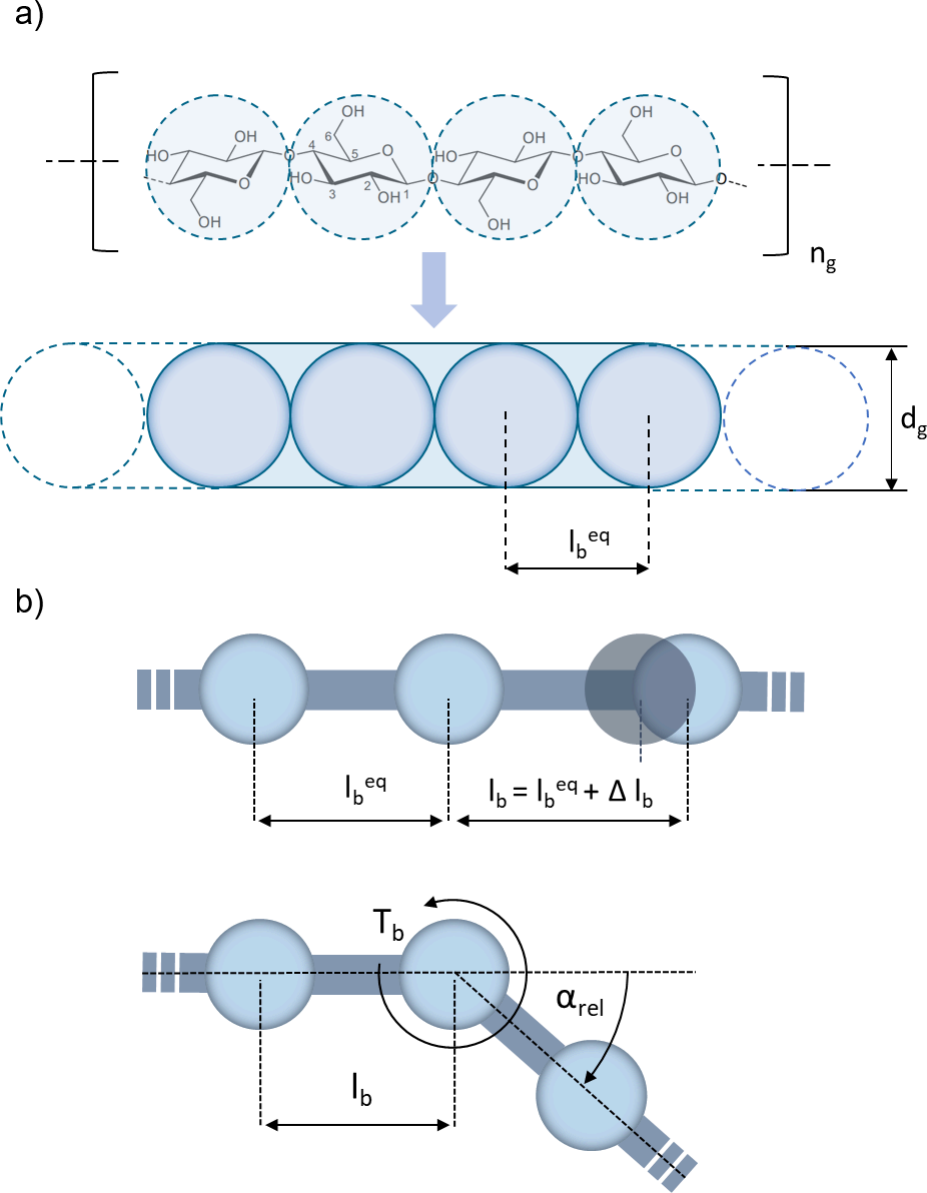
Schematic representation of a single biopolymer (cellulose) linear structure and geometry of d-glucose repeating units (a) and schematic visualization of normal and bending degree of freedom of the polymer bond model (b).

The DEM approach in this work aims at modeling complex systems with simplified discrete spheres (chain-of-beads structure). The focus of the developed model lies in the description of cellulose II gelation, which lacks the crystallinity typically observed in cellulose I. In the studied case, a single sphere represents the most basic structural repeating unit of the polymer (cellulose)–the d-glucose molecule. Cellulose is a linear polysaccharide biopolymer consisting of *n*_g_d-glucose molecules connected with 1,4-β-glycosidic bonds. A schematic representation of a biopolymer structure is presented in Figure 2a.

Each of the glucose repeating units is represented as a sphere with a constant diameter *d*_g_ and mass *m*_g_. The diameter of the spheres is selected as equal to the length of d-glucose molecule, *d*_g_ = 0.4615 nm, and the same value is chosen as an equilibrium distance between the sphere’s centers *l*_b_^eq^ = 0.4615 nm. The mass of a single sphere is established based on a molar mass (*M*_g_ = 162.14 g mol^–1^) of the glucose *m*_g_ = *M*_g_/*N*_A_ = 2.692 × 10^–22^ g, where *N*_A_ is the Avogadro number. The number of the d-glucose repeating units is selected based on experimental raw cellulose properties chosen for the model validation. The experimental value for the degree of polymerization refers to the number of cellobiose repeating units per polymer chain. These cellobiose monomers each consist of two glucose repeating units rotated around the polymer chain axis about 180°. This results in the average number of d-glucose repeating units *n*_g_ = 2·*D*_P_ = 360, leading to the final length of a single cellulose chain equal to *l*_c_ = 415.35 nm.

### Functional Model

The motion of the d-glucose units is induced by the resultant force acting upon them. The forces are derived by accounting for the diffusion (), bond elasticity () and intermolecular interaction between molecules located within the cutoff distance (). In this case, the Newton equation of motion takes the form of:
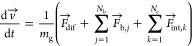
3where  is the vectorial velocity of a monomer, *N*_b_ is the number of bonds connected to a particular d-glucose repeating unit with *N*_b_ ∈ [1,2], and *N*_c_ is the number of d-glucose repeating units of other cellulose chains located within an interaction distance *d*_int_ ≤ 1.5 nm. By using a leapfrog algorithm, the position and velocity of a given d-glucose unit can be followed through the simulation with the time step d*t* = 0.04 ps, which has proven to be the largest possible time step to obtain converging simulation results for the system. The position components *r*_*i*_ and velocity components *v*_*i*_ in the three directions of space (*i* ∈ {*x*, *y*, *z*}) of every d-glucose repeating unit are calculated via eqs 4 and 5.
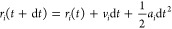
4
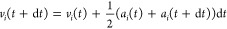
5

The restoring force acting upon the chain segments () arises from the linear elasticity within the polymer bonds and is responsible for chain elongation and curvature. The interaction between adjacent repeating units within one chain is described by a linear elastic model with two degrees of freedom–normal elongation and bending of the polymer bond (presented schematically in Figure 2b).^13^

The 1,4-β-glycosidic bonds between the d-glucose units (represented by spheres, connected with cylinders) are assumed to act like Hookean springs in the chain direction with the spring constant *k*_b_^*n*^. The scalar value of the normal bond force component (associated with the bond elongation, the normal degree of freedom) is calculated in eq 6 and acts in the direction of the bond axis between two neighboring d-glucose units of the same cellulose chain.
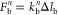
6

The spring constant is estimated based on experimentally determined values of the stiffness (*s* = 40.7 pN) and persistence length (*l*_p_ = 6.2 nm) of a single cellulose molecule.^14^ The experimental chain stiffness, which is approximated by an elastic rod of length *l*_p_, was converted into normal stiffness of the polymer bond, by considering the cross-sectional area of the modeled chains, leading to the final value *k*_b_^*n*^ = (*l*_p_*s*)/*l*_b_^2^ = 1.18479 N m^–1^. The ratio *l*_p_/*l*_b_ is the number of spring elements or the number of bonds in a cellulose chain of length *l*_p_.

The scalar value of the bending torque is established by the linear elastic law in eq 7 where *k*_b_^α^ is the bending stiffness and α is an angle created by two adjacent bonds.
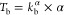
7

The bending torque results in a force acting on three glucose units that are connected by the two given bonds (as presented in Figure 2a). The obtained bending stiffness value is *k*_b_^α^ = 1.15375 × 10^19^ N m rad^–1^, which was established in concordance with^9^ to obtain similar chain flexibility.

The diffusion model is implemented as an external field model in the MUSEN framework. It accounts for the characteristic physicochemical properties of the system, such as interaction between the NaOH-urea aqueous solvent and cellulose chains. The classic Langevin dynamics was simplified by considering only isotropic translational diffusion and omitting the aspect of rotational diffusion,^15^ as this DEM approach considers diffusion of isotropic spheres.^9^ The forces for translational diffusion acting on the repeating units for each degree of freedom *i* ∈ {*x*, *y*, and *z*} are described by eq 8, where *v*_*i*_ is the velocity component of a d-glucose monomer in the respective direction of space and ξ_*i*_ is a random number generated based on a normal distribution. The dissipative drag coefficient *c*_*i*_ is defined by eq 9, where μ_s_ = 0.03 Pa s is solvent dynamic viscosity (the value was measured experimentally), *r*_s_ = 0.23075 nm is the Stokes radius of the glucose repeating unit, *k*_b_ is the Boltzmann constant, *T* = 300 K is the temperature of the system, and Δ*t* is the simulation time step. The calculated Stokes radius coincides with published experimental data.^16^ The fluctuating force components *F*_f,*i*_ are calculated according to eq 10.
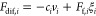
8
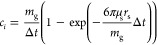
9
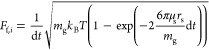
10

The intermolecular interaction between repeating units is implemented as a particle–particle contact model in MUSEN, based on the Lennard-Jones potential. The scalar value of the interaction force *F*_int_, which acts in the direction of the connecting vector between the centers of two d-glucose repeating units, is calculated via eq 11.
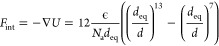
11where ϵ is the depth of the potential well, *N*_a_ is the Avogadro number, *d* is the time-dependent distance, and *d*_eq_ is the equilibrium distance, i.e., the location of the minimum of the Lennard-Jones potential. This model describes both attractive and repulsive interaction; however, it is possible to define the interaction as purely repulsive, for example for the equilibration step to minimize the overlap between glucose repeating units in the RVE. Furthermore, for limiting computational cost, a cutoff distance of 1.5 nm is used according to ref (17).

The parameters for the polymer bond model and the diffusion model are physically motivated, as described above. In order to explore the model’s boundaries and behavior and the influence on the resulting microstructure, the interaction model was chosen for a parameter sensitivity analysis. The range of studied values is ϵ ∈ ⟨5.0; 42.0 kJ mol^–1^⟩ and *d*_eq_ ∈ ⟨0.3; 0.425 nm⟩.

### Simulation of Gelation

The simulation has a sequential character, consisting of the following steps: system generation, relaxation, equilibration, and gelation. The computational representation of the subsequent treatment of the obtained gel structure (washing and solvent exchange) is described in the next subsection.

The first step is system generation—for the defined RVE with periodic boundary conditions, straight cellulose chains (consisting of *n*_g_ = 360 as a number of repeating d-glucose units) are generated with random spatial orientation. The number of generated cellulose chains inside the volume of the simulation domain *V*_RVE_, *n*_c_ = 764, was derived based on the weight percentage *w*_c_ of cellulose and the density ρ_sol_ of the gelling solution, according to
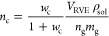
12

The next step is the relaxation of the straight chains to resemble the real nature of the dissolved cellulose chains in aq. NaOH-urea solution. During the relaxation step (15 μs), the diffusion and polymer bond models are activated, aiming at obtaining natural, relaxed state of the chains. The relaxation is supported by an adapted procedure of annealing presented in ref (9). The temperature of the system is artificially increased to *T*_max_^a^ = 2000 K and subsequently decreased in a linear manner within the time interval of τ = 5 μs to the equilibrium temperature *T*_eq_ = 300 K.

Subsequently, the equilibration step is performed (700 ns), where in addition to the polymer bond and the diffusion model, the repulsive Lennard–Jones interaction is activated in order to correct and minimize the previously generated overlap of d-glucose molecules. The system after these three steps (generation, relaxation, and equilibration) represents the solute system of cellulose molecules in aq. NaOH-urea solution and represents the starting point for the gelation.

**Figure 3:**
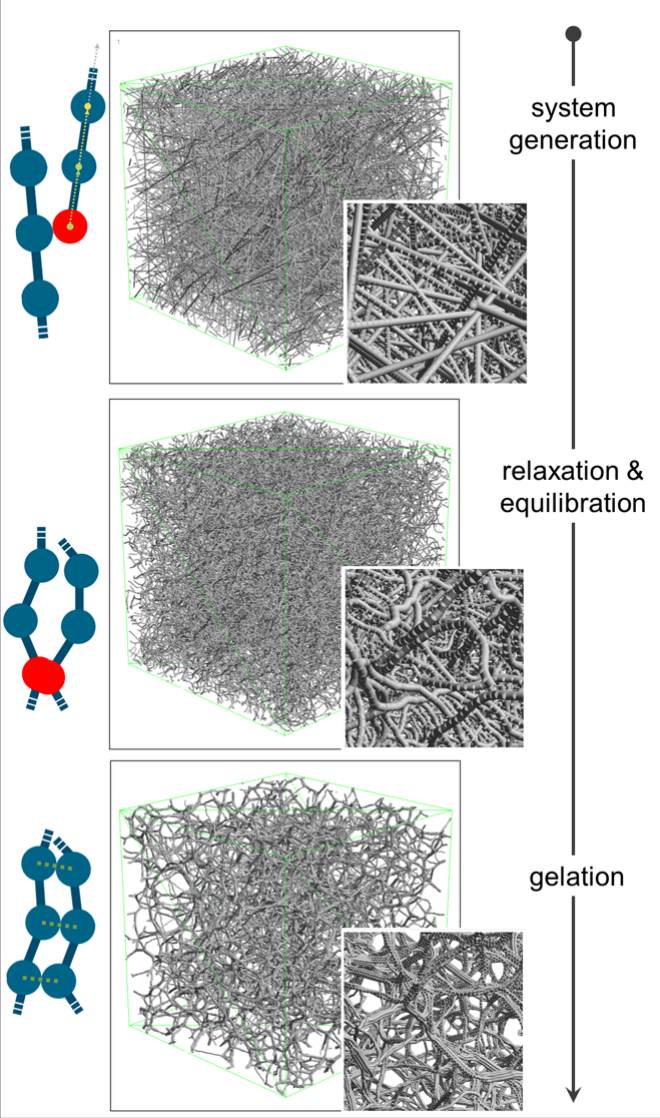
Visualization of the simulation procedure for structure generation.

During the gelation step, all of the model components are active (polymer–bond, diffusion, and both attractive and repulsive interaction models). The gelation step is performed for 5 μs, as after this time, the connectivity between the chains did not change significantly. The overall scheme of the virtual production pipeline is presented in Figure 3.

### Simulation of Washing and Solvent Exchange

According to the experimental procedure, the next step of the computational approach is to simulate washing the obtained wet gel (the washing step) with water and, subsequently, with ethanol (the solvent exchange step), leading to obtaining the final gel product before supercritical drying—the alcogel.

The process was computationally implemented by changing the viscosity of the solvent in a system, according to real values for water and ethanol at room temperature (μ_w_ = 0.00089 Pa s and μ_e_ = 0.001074 Pa s, respectively^18^). This numerical procedure highlights the influence of viscosity on the diffusion and self-reorganization of the formed cellulose chain bundles and, thus, the geometry of the pores.

### Simplistic Model for Gel to Aerogel Transition

Supercritical extraction of the solvent filling the pores of a gel, carried out in an autoclave, is critical for preserving the original structure of the sample subjected to the drying process. However, even when using this advanced drying technique, volume shrinkage should be accounted for. The deformation of the structure is dependent on the pore geometry and the capillary pressure inside them; moreover, any deviations of the drying conditions (such as temperature or pressure inside the autoclave) can lead to uneven stress distribution and not predictable results, which makes developing a reliable, physical model a computational challenge. To simplify the drying process in this first study, isotropic deformation mimicking the isotropic shrinkage arising from the pressure subjected to within the autoclave is applied on the gel network. The deformation applied is based on the volumetric shrinkage observed in the experiments. The deformation gradient tensor for isotropic deformation (denoted as *F*) is defined as
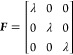
13where λ is the linear stretch, reflecting the linear shrinkage calculated based on the value of experimentally observed volumetric shrinkage (39.5%) as follows: λ = *l*/*L* (with  denoting the deformed length and *L* is the original characteristic length).

This approach artificially accounts for the expected volume shrinkage of the structure and its influence on the pore volume, allowing for better validation of the developed DEM approach potential for representation of the biopolymer-based aerogel system.

### Postprocessing

The microstructural characterization of the generated cellulose gel structure and the comparison with experimental data require several postprocessing steps. The Cartesian coordinates of the glucose repeating units constituting the cellulose polymer chains are voxelized based on a 0.25 nm discretization using the *Open3D Python* library.^19^ The resulting three-dimensional binary image indicates solid regions (*True* values) and pore regions (*False* values) of the virtual microstructure.

**Figure 4:**
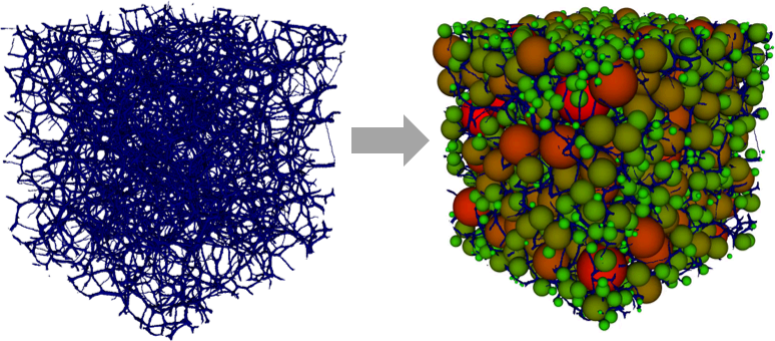
Three-dimensional binary image of generated cellulose aerogel microstructure (left) and pores of the extracted pore network model inside the binary image (right).

In order to extract a pore network model from the voxelized binary image, the *SNOW* algorithm developed by Gostick^20^ is applied. The extracted pore network model, which is compatible with the *OpenPNM*(21) pore network modeling package, consists of spherically defined pores that are connected with cylindrical throats. The binary image and pores of the extracted pore network model are depicted in Figure 4.

The pore-size distribution of the experimental validation material is of a discrete nature, whereas the computationally generated pore sizes are continuously distributed. The simulative distribution is therefore discretized based on a moving window approach under consideration of the given boundary values of the experimental measurement data. The respective pore volumes for both experimental and computational data are approximated under consideration of a spherical pore shape using the average pore diameters *d*_P,av,*i*_ of each discretization window *i*. The incremental pore volume of the computational model can be expressed as
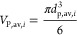
14

Conversely, to compare the statistical characteristic values of the pore-size distributions, the discrete experimental distribution is converted to a continuous distribution. Here, an array of pore diameters is generated by extracting the frequency of the respective pore widths. The experimental pore volume *V*_P,tot_ contained by the macroscopic cellulose aerogel specimen is correlated with the volume *V*_rve_ of the simulation domain. This step requires a downscaling of the experimental pore volume under the assumption that the percentage of pores occupying a specific fraction of the total pore volume, *V*_P,tot_ is consistent throughout the length scale. The experimental frequency *n*_P,*i*_ for each discrete average pore width *d*_P,av,*i*_ is calculated as
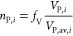
15where *V*_P,*i*_ is the respective experimentally measured incremental pore volume for a discrete *d*_P,av,*i*_ value and *f*_V_ is the volume scaling factor, considering the experimental porosity Φ_exp_ of the cellulose aerogel specimen:
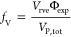
16

These steps and assumptions enable a comparison of the pore-size distributions based on characteristic statistical values.

The porosity of the computationally generated microstructure is derived from its binary image representation. With *n*_pore_ referring to the image voxels indicating pore regions and the total number of voxels of the image *n*_tot_, the porosity of the modeled microstructure is calculated as
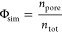
17

## Results and Discussion

### Physical Properties from Experimental Data

The volume shrinkage of the cellulose aerogel beads is 39.5% (using eq 1), agreeing with the data which were previously reported in the literature.^11,22,23^

The skeletal density of cellulose aerogel beads is 1.52 g cm^–3^, which closely resembles the values reported in the literature.^11,24,25^ The envelope density and porosity values are shown in Table 1. Both values are within the range mentioned for cellulose aerogels.^11,26^ The tapping density analysis showed a value of 0.11 g cm^–3^. The porosity of cellulose aerogel beads is calculated by using eq 2, which is about 85%.

**Table 1:** Physical Properties of Cellulose Aerogel Beads

envelope density/g cm^–3^	porosity/%	BET specific surface area/m^2^ g^–1^	BJH average pore diameter/nm	BJH total pore volume/cm^3^ g^–1^
0.23 ± 0.01	85	379 ± 2	28.9 ± 0.3	3.67 ± 0.01

**Figure 5:**
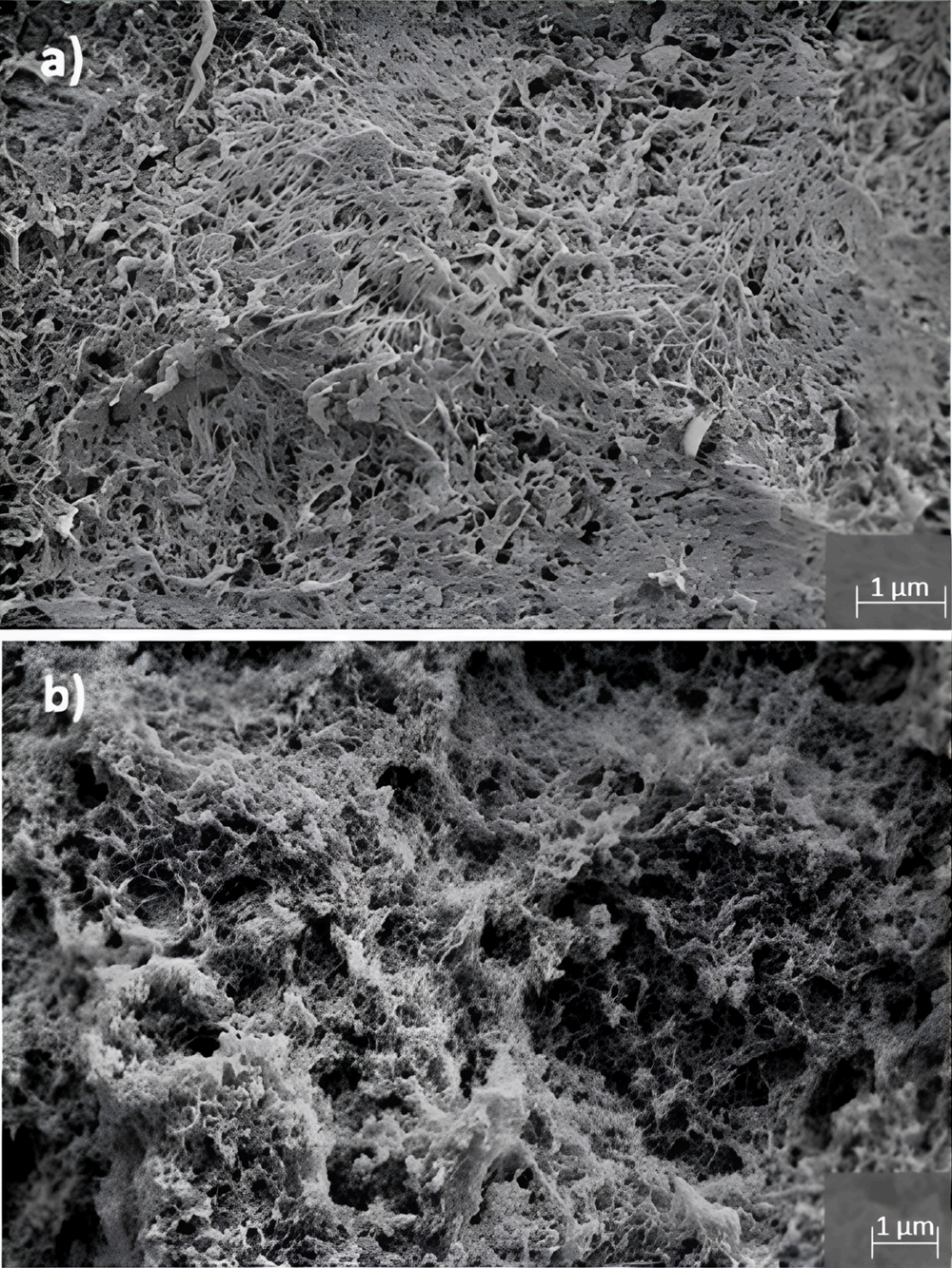
SEM images of cellulose aerogel beads: (a) microstructure of the surface and (b) inner microstructure after fracturing the beads.

Figure 5 shows the structure of the surface morphology as well as the inner structure of the cellulose beads. Both images show the randomly arranged interconnected nanofibrillar network and the open porous structure which are characteristics of cellulose aerogels.^11,27,28^

**Figure 6:**
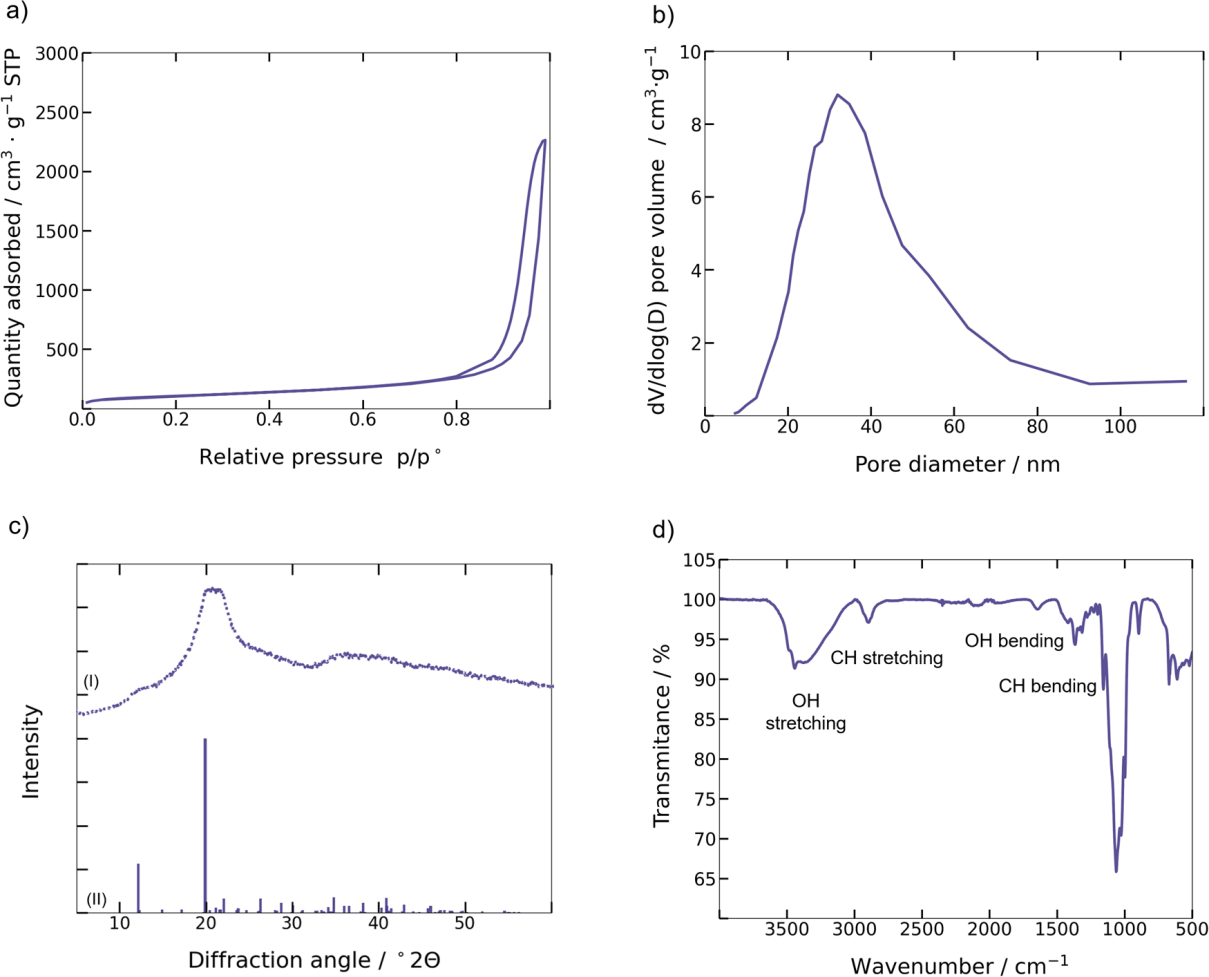
Properties of cellulose aerogel beads from (a) nitrogen adsorption–desorption isotherm, (b) BJH pore size distribution, (c) X-ray diffraction data (I) in which the vertical lines at the bottom (II) indicate the reference diffraction pattern of cellulose II (PDF = 00–056–1717) obtained from International Center for Diffraction Data, and (d) ATR-FTIR spectrum.

Figure 6a shows a representative nitrogen adsorption–desorption isotherm for the cellulose beads. The progression of the isotherm is characteristic of a type IV isotherm as defined by the IUPAC classification.^29^ The well-pronounced hysteresis in the isotherm is attributed to mesoporous materials.^27,30^

The BET specific surface area, the BJH average pore diameter, and the BJH total pore volume of the cellulose beads are shown in Table 1. The results show a good agreement with the data reported in literature.^11,25,27,31^ The BJH pore-size distribution shows the existence of a larger number of mesoporous structures and only a smaller number of macropores (Figure 6b). The average pore diameter is within the range for mesoporous materials (2–50 nm).

Figure 6c shows the powder X-ray diffraction spectrum of the cellulose aerogel beads. It possesses the crystalline configuration of cellulose II as the molecular chains align in an antiparallel way during gelation. The major peaks at ∼12.5 and 20° correspond to the crystalline plane 1̅10 and 110, respectively, which are assigned according to the diffraction pattern (PDF number = 00–056–1717) reported in the International Center for Diffraction Data. The broad diffraction pattern indicates that the aerogel beads could have a mixture of poorly crystalline cellulose II and amorphous cellulose.

The FTIR spectra of the cellulose aerogel beads are shown in Figure 6d. The broad peak from 3000 to 3700 cm^–1^ is assigned to symmetric and asymmetric OH stretching of inter- and intramolecular hydrogen bonds in cellulose. The peak at ∼2894 cm^–1^ represents CH- stretching in polysaccharides. The absorption band at ∼1633 cm^–1^ corresponds to the −OH bending vibration of the adsorbed water molecules in cellulose. Furthermore, the bands at ∼1422 and ∼1368 cm^–1^ are associated with CH_2_ and CH bending vibrations in cellulose. Comparing the literature data, it can be concluded that synthesized cellulose aerogel beads do not have any contaminations or noncellulose components.^32,33^

### Gelation Simulation Results

The virtual cellulose aerogel structure is generated during the last step of the simulation sequence, namely, the gelation. During computational gelation, the individual cellulose chains begin to interact with one another. The interaction between two adjacent cellulose polymer chains modeled with the proposed DEM-based gelation model also exhibits the commonly observed zipper-like aggregation behavior,^34,35^ which is schematically and sequentially illustrated in Figure S.1 in the Supporting Information.

**Figure 7:**
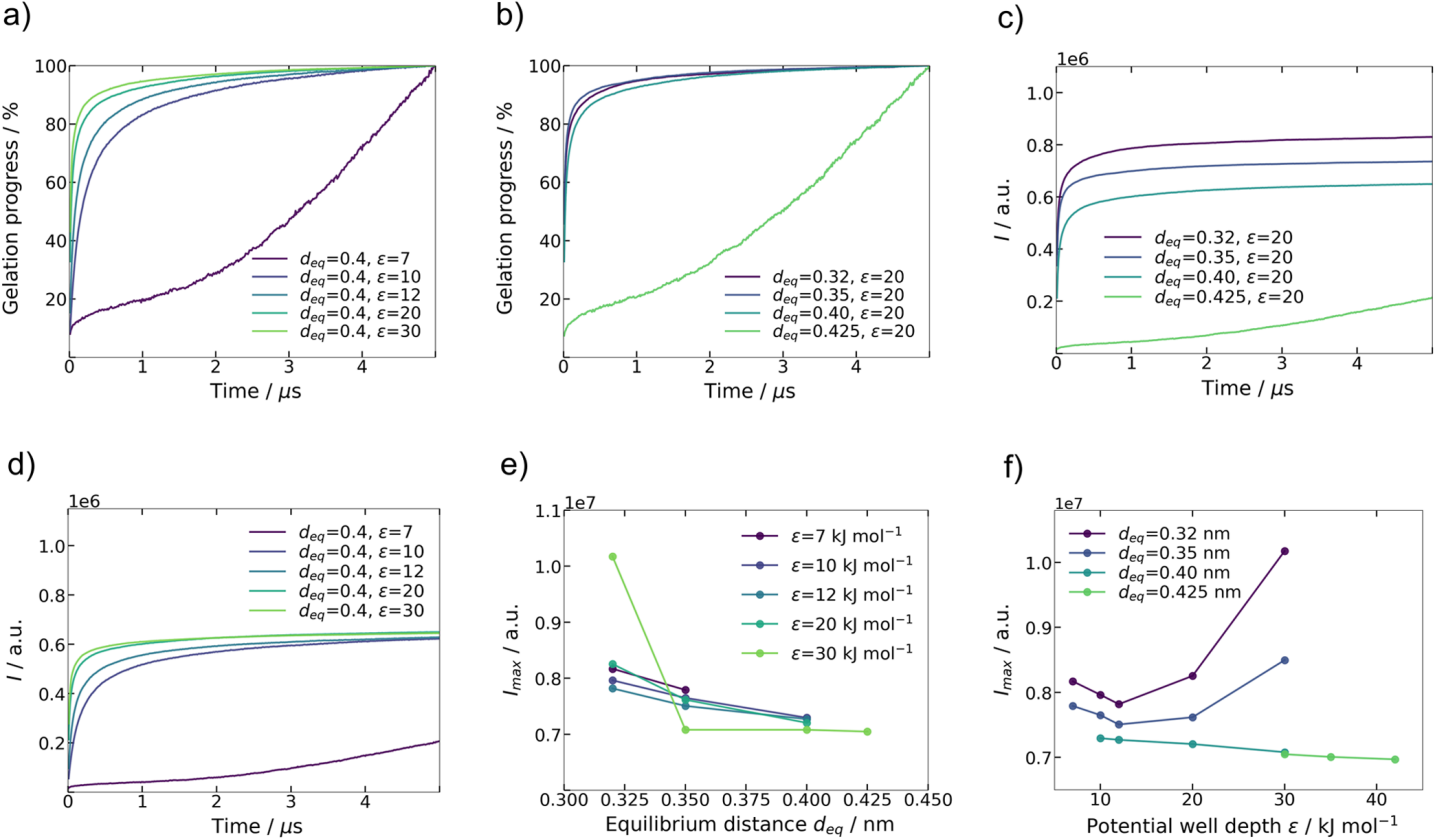
(a) Virtual gelation progress (*I*/*I*_max_ in %) for constant *d*_eq_ = 0.4 nm, (b) virtual gelation progress (*I*/*I*_max_ in %) for constant ϵ = 20 kJ mol^–1^, (c) intermolecular interactions (I) between glucose repeating units of the cellulose polymer chains during gelation simulation for constant ϵ = 20 kJ mol^–1^, (d) intermolecular interactions (I) between glucose repeating units of the cellulose polymer chains during gelation simulation for constant ϵ = 20 kJ mol^–1^, (e) maximum intermolecular interactions *I*_max_ between glucose repeating units of the cellulose polymer chains at τ = 5 μs as a function of *d*_eq_, and (f) maximum intermolecular interactions *I*_max_ between glucose repeating units of the cellulose polymer chains at τ = 5 μs as a function of ϵ.

The development of the number of intermolecular interactions between glucose repeating units as a function of simulation time τ is significantly influenced by the parameters ϵ and *d*_eq_ of the Lennard-Jones potential. The gelation progress during simulation is visualized in Figure 7a,b based on the normalized number of intermolecular interactions *I*/*I*_max_ between glucose repeating units of the cellulose polymer chains. *I*_max_ refers to the maximum number of interactions between d-glucose repeating units in the entire simulation domain at the end of the gelation simulation. The respective *I*_max_ values can be deduced from Figure 7c,d at τ = 5 μs. The Lennard-Jones parameter combinations *d*_eq_ = 0.425 nm, ϵ = 20 kJ mol^–1^ and *d*_eq_ = 0.4 nm, ϵ = 7 kJ mol^–1^ do not result in an aggregated network of the cellulose polymer chains. Hence, no fully gelled microstructure is generated for these two cases. This distinction is also noticeable in the markedly different shapes of the gelation kinetics associated with these two parameter sets in Figure 7a–d. The remaining simulated combinations for ϵ and *d*_eq_ which were chosen for the interaction model within the scope of this work result in a gelled, fibrillar network of cellulose polymer chains that visually exhibits resemblance with experimentally observed microstructures. The open-porous nature of cellulose aerogels is successfully generated with these parameter combinations. Postgelation morphology and coordination number visualization for two representative cases: (i) system considered as gelled successfully (*d*_eq_ = 0.4 nm, ϵ = 30) and not successfully (*d*_eq_ = 0.4 nm, ϵ = 7) are included in the Supporting Information, Figure S.2. The coordination number of one d-glucose repeating unit is defined as the number of the direct interactions with other d-glucose repeating units.

Figure 7a indicates a correlation between the interaction potential well depth ϵ and the shape of the gelation kinetics. The cellulose aggregation occurs faster with increasing ϵ. Two glucose units from separate cellulose chains maintain cohesion if the molecular interaction forces between their respective glucose repeating units surpass the intramolecular forces from inside the polymer chain and the diffusion forces influencing these glucose units. The fact that ϵ directly scales the interaction forces elucidates the accelerated gelation kinetics for larger ϵ values.

On the other hand, intermolecular interaction forces increase with decreasing equilibrium distance *d*_eq_. Here, no clear correlation between the parameter and gelation kinetics can be drawn from Figure 7b. However, for a given potential well depth ϵ, a maximum equilibrium distance *d*_eq_ exists, representing the upper limit for successful gelation simulation. Similarly, a minimum potential well depth ϵ exists as a lower limit for a given equilibrium distance *d*_eq_.

The exact values for the limits of gelation depend on the parameters of the subparts of the functional model, i.e., diffusion model and polymer bond model.

Figure 7c,d visualize the total number of intermolecular interactions during the gelation simulation as a function of the virtual simulation time τ. In Figure 7c, for a constant ϵ, decreasing *d*_eq_values result in an increase of total intermolecular interactions at τ = 5 μs. This trend is valid for all simulated values for ϵ. In Figure 7d, it is not possible to derive a similar trend for variable ϵ and constant *d*_eq_. Figure 7e indicates an inverse proportionality between *d*_eq_ and the maximum number of intermolecular interactions *I*_max_ at τ = 5 μs. The influence of *d*_eq_ is more pronounced for a larger ϵ. One possible explanation for the influence of the Lennard-Jones equilibrium distance is the denser packing and increased overlap of cellulose polymer chains inside aggregated bundles for smaller *d*_eq_. This causes more glucose-repeating units to simultaneously interact with one another.

*I*_max_ as a function of ϵ is depicted in Figure 7f. For the simulated parameter space, it is difficult to obtain correlations between *I*_max_ and ϵ. However, it can be stated that for *d*_eq_ = 0.32 nm and *d*_eq_ = 0.35 nm, *I*_max_ increases for larger ϵ.

Undoubtedly, the complex behavior of the computational gelation model is influenced by the parameters of the interaction model. The intermolecular forces between modeled glucose repeating units increase with increasing ϵ and decreasing *d*_eq_. However, for the simulated system of gelling cellulose, several cellulose polymer chains simultaneously interact. The same chain may contribute to the formation of several molecule bundles forming the fibrillar cellulose network. This is very likely considering the length of the cellulose polymer chains with respect to the dimensions of the RVE. Furthermore, the stiffness properties and local curvature of the polymer chains and the resulting intramolecular forces influence the computational gelation characteristics. These facts make it difficult to draw a definite conclusion with respect to the influence of the Lennard-Jones potential parameters on the gelation kinetics based on a physically motivated explanation.

From the simulated parameter set for the interaction model, it is recognizable that the chosen values for the equilibrium distance *d*_eq_ are unanimously lower than the diameter *d*_g_ of the d-glucose repeating units, leading to the interpenetration of the repeating units. For the physically motivated chosen set of parameters in the bond model and the diffusion model, larger chosen equilibrium distances currently prohibit gelation of the virtual system of cellulose polymer chains. Owing to the complexity of the model, further investigations exploring the limits of gelation and the effect of modified parameters in the polymer bond and diffusion model are recommended.

**Figure 8:**
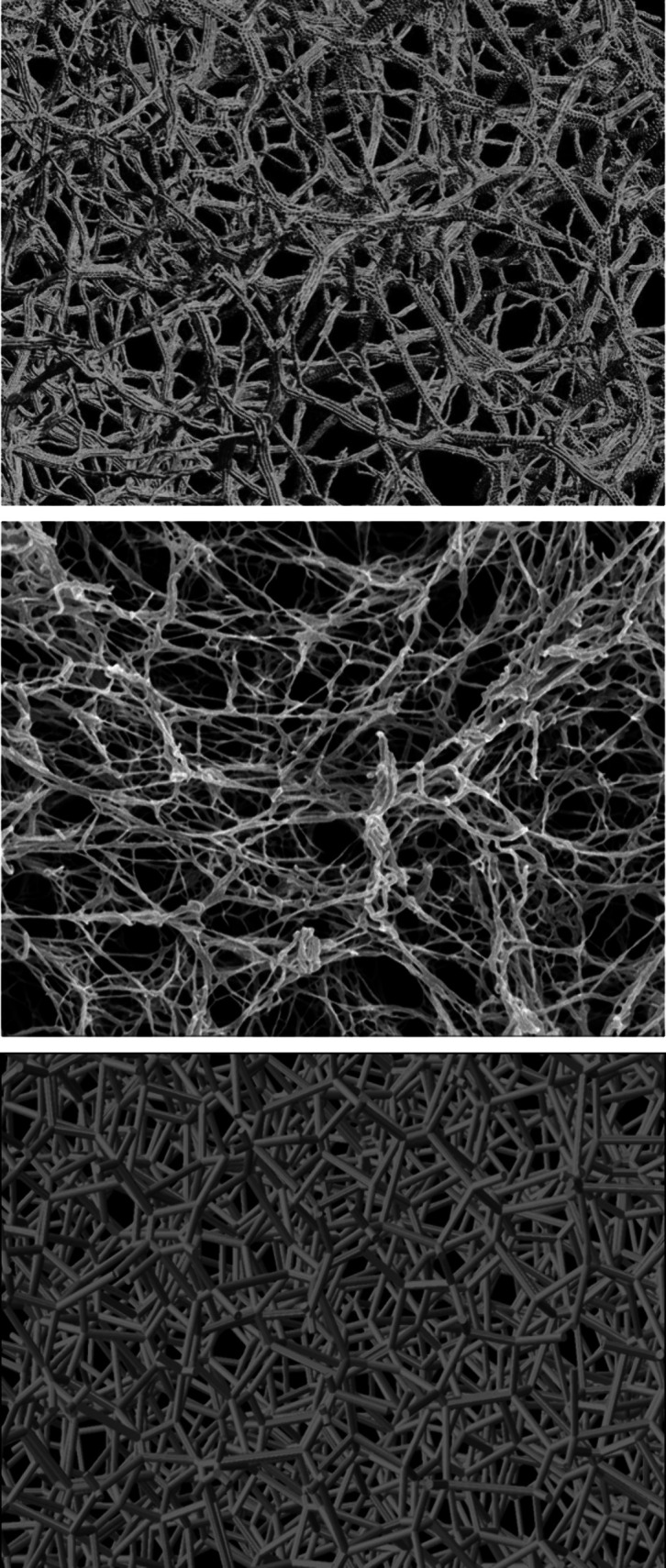
Comparison of aerogel microstructure generated with DEM gelation model (top) with SEM-image of cellulose aerogel^25^ (center) and reconstructed microstructure using Voronoi approach (bottom).

This research aims to prove the suitability of the DEM-based, coarse-grained model approach for mimicking the gelation process of cellulose with a focus on microstructure generation and comparison with experimental data. Figure 8 provides a juxtaposition of the virtual cellulose (aero)gel fibrillar network, an SEM image of a cellulose aerogel sample, and a reconstructed aerogel microstructure via Voronoi tessellation (approach used in previous work of Aney and Rege^36^). With the proposed sequential approach for computational biopolymer gel generation, an adequate representation of the cellulose wet gel is successfully obtained. The simulated structure exhibits a great visual similarity with experimental SEM images of cellulose aerogels, especially with respect to their fibrillar microstructure.

The schematic comparison presented in Figure 8 aims to demonstrate the similarity in the types of morphologies resulting from both experiments and simulations. The SEM image of the original sample, which was used for validation of our model, is presented in Figure 5, and one can observe that the scale of the fibrillar structure is comparable to the one produced in the simulations.

Compared to Voronoi tessellation approaches, the gelation model in this work offers enhanced capabilities for capturing these characteristic fibrillar structures. It is crucial to note that, in contrast to Voronoi tessellation methods, which rely on experimental microstructure data to reconstruct a computational virtual twin, the DEM-based gelation model can generate a virtual representation of the desired microstructure by simulating the aggregation and network formation during the gelation process on a molecular level.

**Figure 9:**
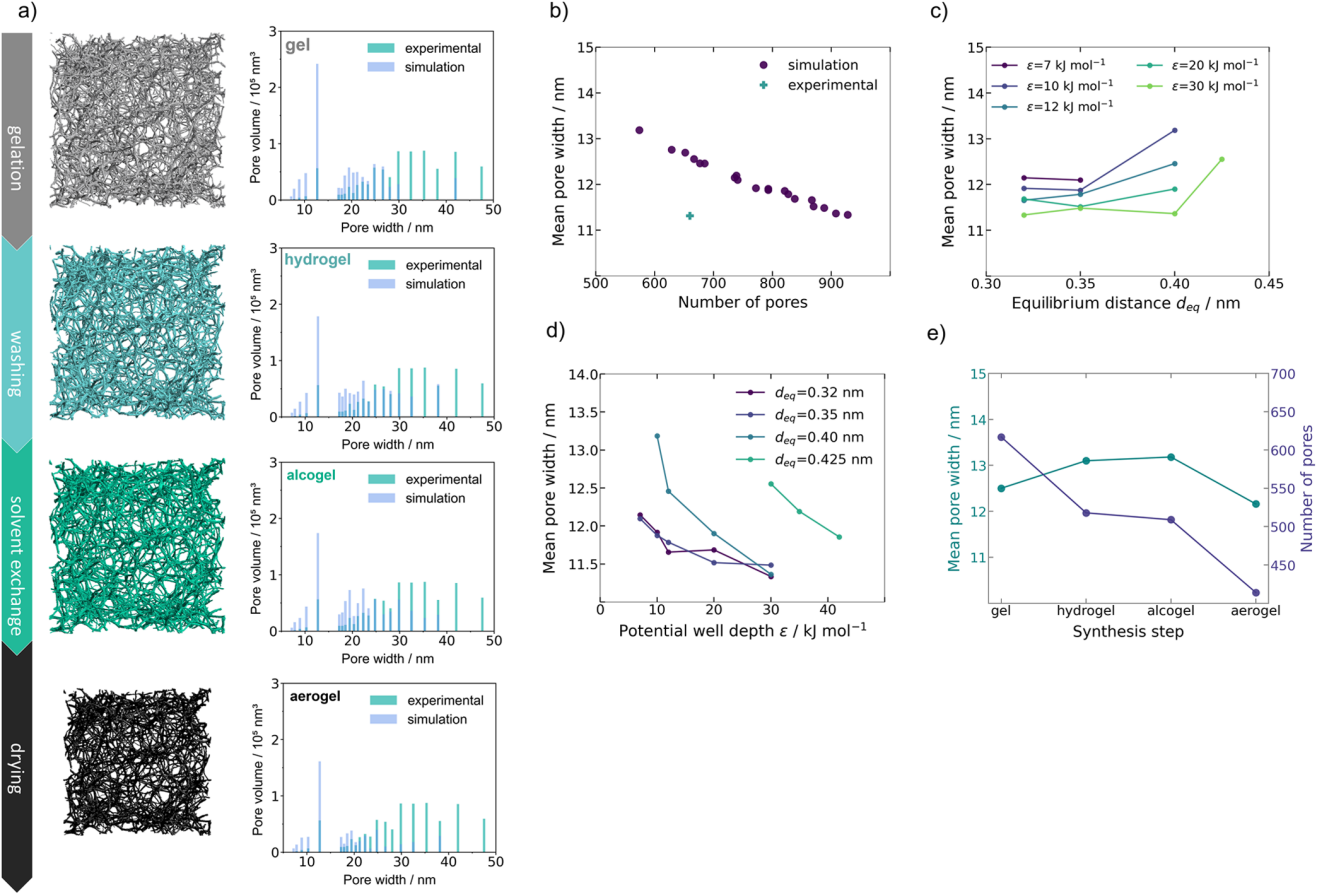
(a) Dependence of the synthesis steps within the model on pore size distribution, (b) correlation of number of pores *n*_*P*_ and mean pore width *d̅*_P_ of computational gelled microstructure, (c) mean pore width *d̅*_P_ of the computational microstructure as a function of the Lennard-Jones potential parameter *d*_eq_, (d) mean pore width *d̅*_P_ of the computational microstructure as a function of the Lennard-Jones potential parameter ϵ, and (e) increase of mean pore width and decrease in the number of pores depending on the steps of synthesis of the numerically gelled structure.

The observed range of pore sizes and the mean pore widths *d̅*_P_ of the computationally generated microstructure lie well within the same order of magnitude as the widths of the pores inside the experimental cellulose aerogel specimens. However, it is observed that the larger pores of the experimental specimens with a width of *d*_P,av, and *i*_ > 30 nm were not captured by the gelation model for cellulose. This is owing to the simulation box size (RVE) limitation. Figure 9a illustrates the evolution of incremental distribution of the pore diameter *d*_P,av,*i*_ with the steps of synthesis (gelation, washing, solvent exchange) considering the respective contribution to the pore volume *V*_P,av,*i*_ for the Lennard-Jones parameter combination *d*_eq_ = 0.425 nm and ϵ = 20 kJ mol^–1^ for the interaction model.

Figure 9b visualizes the correlation between the number of pores *n*_P_ and the mean pore width of the pore network model extracted from the generated microstructure after gelation. The comparison with the experimental values indicates that the gelation model marginally overestimates the number of pores *n*_P_ for concordant mean pore widths *d̅*_P*,*_ while also overestimating the mean pore width *d̅*_P_ for matching *n*_P_ values. A correct prediction of *d̅*_P_ is of higher significance with respect to the microstructure characterization than a correct prediction of *n*_P_. However, there remains potential for further improvement of the gelation model to predict *d̅*_P_ and *n*_P_ with higher accuracy, for example, by increasing the RVE size, extending the model with rotational diffusion, and considering the drying and shrinkage effects on the final porous structure.

The two Lennard-Jones potential parameters of the interaction model influence the pore size distribution characteristics. While there is no definite trend derivable for the influence of *d*_eq_ for the simulated parameter space, it is visible from Figure 9c that *d*_eq_ ≥ 0,4 nm has increased mean pore widths *d̅*_P_ as a result for all simulated potential well depths ϵ. Similarly, the *d̅*_P_ values decrease for increasing ϵ values for the simulated equilibrium distances *d*_eq_, as shown in Figure 9d. It should be noted that a possible bias due to the postprocessing and the assumption of spherical pore shapes cannot be completely ruled out at this point. Furthermore, a broader-based parameter sensitivity study considering not only the intermolecular interaction model but also the polymer chain bond model and the diffusion model may yield beneficial insights into the model behavior and its capabilities with regard to the reduction of experimental efforts and reverse materials engineering approaches.

The analysis of the obtained hydro- and alcogel structures in terms of pore-size distribution reveals a shift of the pore-size distribution toward wider pores, followed by pore size reduction during drying. This tendency, along with morphologies of aerogel as well as the intermediate products (gel, hydrogel, and alcogel) is schematically presented in Figure 9a. The term “gel” refers to the initial state of the material, which is a wet gel filled with the mother liquid, i.e., a mixture of unreacted or residual compounds from the preparation process, including NaOH, urea, acetic acid, and water. It is distinguished from the “hydrogel” state, which is obtained after the gel has been immersed in pure distilled water, resulting in the removal of the original mother liquid. Subsequently, the “alcogel” refers to the state of the gel after a solvent exchange process, where water is replaced with ethanol. Finally, the “aerogel” corresponds to the dried state of the material, where the pores are filled only with air. Simulation reveals that the mean size of a pore increased by 4.8% after washing with water and, subsequently, by 0.6% after solvent exchange to ethanol. The initial increase was followed by the volume shrinkage occurring during the drying step, leading to a 7.7% decrease in the mean pore width. Correspondingly, during the postprocessing of the wet gel, the number of pores decreases significantly: 16% after washing, another 1.7% after the solvent exchange, and a further 18.7% with drying. The reduction in the number of pores due to isotropic shrinkage could be associated with structural rearrangements. Thus, washing seems to have a strong effect on the pore structure evolution during the synthesis of the aerogels. While the origin of macropores in cellulose aerogels is not fully known, structural rearrangements during washing and solvent exchange seem to open up larger pores. While this effect was observed over several simulations, this demands further investigation on this matter, perhaps by simulating over larger domain sizes and employing state-of-the-art experimental methods to characterize the network in situ. The last step, representing the effect of drying in a simplified manner, led to the promising agreement of experimental and numerical mean pore width (11.31 and 12.16 nm respectively). The character of the observed tendencies is presented in Figure 9e.

## Conclusions

The coarse-grained model proposed in this paper is shown to successfully demonstrate the gelation kinetics in cellulose aerogel systems. In addition, the morphological alterations resulting from the solvent exchange are also simulated. The proposed model is composed of a structural and a functional model. The latter is subdivided further into a bond, interaction, and diffusion model. The diffusion model accounts for the solvent implicitly, thus taking into consideration the importance of the solvent during the diffusion of molecules. The parameters of the interaction model are shown to significantly affect the gelation kinetics. Thus, it becomes essential to identify the interaction model parameters for the desired material system correctly. In its entirety, the proposed model describes the aggregation of the cellulose polymer chains resulting in the formation of fibrils, as well as that of the fibrils forming the 3D porous network. The model predictions align with the experimental results. The RVE size presents the biggest bottleneck while comparing the results, given that the larger pore sizes beyond 30 nm cannot be described with the model owing to the size limitations. To this end, the comparison to macroscopic experimental data remains comparative. The washing and solvent exchange was also simulated. The simulations demonstrate that washing results in a shift in the pore sizes toward wider pores. The mean pore size increased by 4.8% after washing and <1% after subsequent solvent exchange; however, the number of pores reduced by nearly 16% upon washing and further over 1.7% after solvent exchange and 18.7% after drying. This suggests the occurrence of macropores resulting from postprocessing of the formed gel. This needs further investigation by simulating over larger domain sizes. Finally, the drying of the gels was mimicked by subjecting the gel microstructures to isotropic deformation in line with the observed volumetric shrinkage, and the pore structure analysis was presented. Good agreement of experimental and numerical mean pore width values (11.31 and 12.16 nm, respectively) was observed, indicating the clear potential of the developed DEM approach for the representation of biopolymer-based aerogel systems.

## Supporting Information Available

Illustration of the capability of the model to describe the zipper-like mechanism in the aggregation of cellulose chains and two contrasting cases, showcasing the influence of the model parameters on the coordination number and its consequence on the gelation (PDF)
