# Supplementary material for: Modeling of the Gelation Process in Cellulose Aerogels
Source: Biomacromolecules. 2025 Mar 3;26(4):2199–210. doi: 10.1021/acs.biomac.4c01474 (PMC12004520; doi:10.1021/acs.biomac.4c01474)
Supplement: Supplementary file 1 — bm4c01474_si_001.pdf [file bm4c01474_si_001.pdf]

Supporting Information

Modeling of the Gelation Process in Cellulose Aerogels

Jannik Jarms,[†,‡] Nina H. Borzęcka,[†] Bruno Serrador Goncalves,[†] Kathirvel Ganesan,[†] Barbara Milow,[†,¶] and Ameya Rege*[,†,§]

[†]*Institute of Materials Research, German Aerospace Center (DLR), Linder Höhe, 51147 Cologne, Germany*

[‡]*Institute of Mechanics and Computational Mechanics (IBNM), Gottfried Wilhelm Leibniz University Hannover, Appelstraße 9a, 30167 Hannover, Germany*

[¶]*Department of Chemistry, University of Cologne, Greinstr. 6, 50939 Cologne, Germany*

[§]*Department of Mechanics of Solids, Surfaces & Systems, University of Twente, P.O. Box 217, 7500 AE Enschede, The Netherlands*

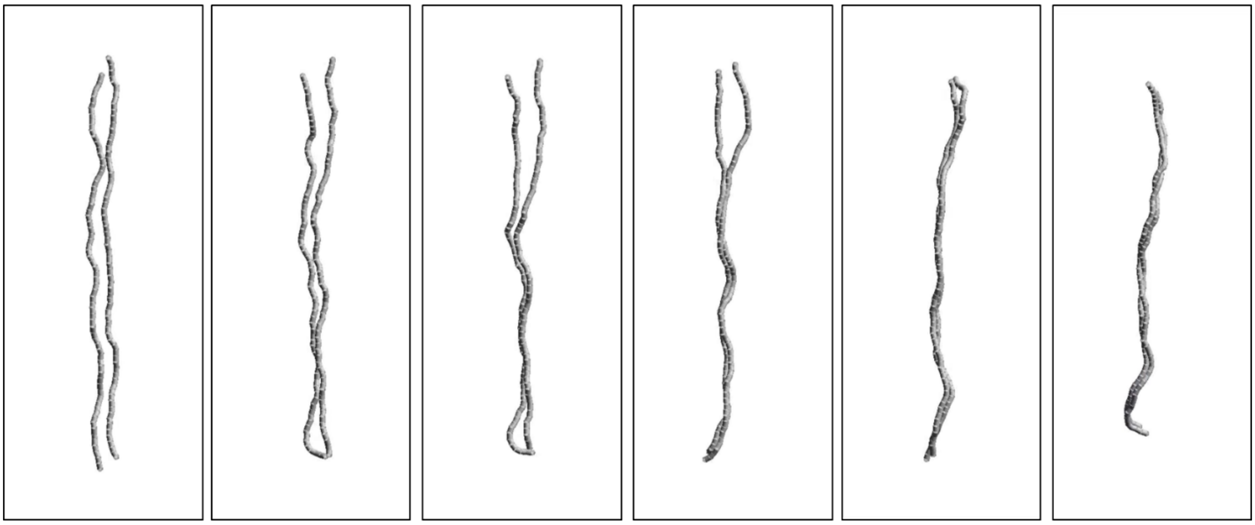

Figure S.1. The progress of the zipper-like aggregation mechanism for the idealized case of 2 adjacent polymer chains (DP=70)

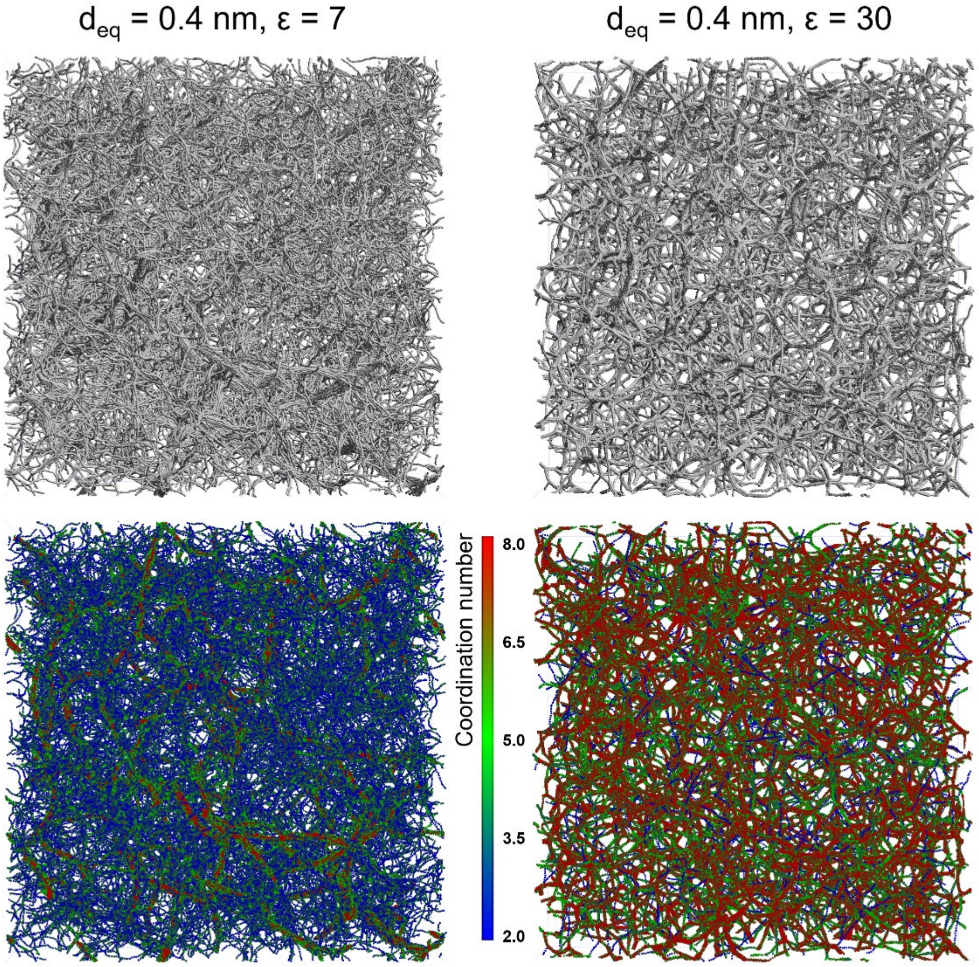

Figure S.2. Post-gelation morphology (upper) and coordination number visualization (bottom) for system after successful ($d_{eq} = 0.4nm$, $\varepsilon = 30$) and not successful ($d_{eq} = 0.4nm$, $\varepsilon = 7$) gelation simulation
